# Markedly Increased High-Mobility Group Box 1 Protein in a Patient with Small-for-Size Syndrome

**DOI:** 10.1155/2014/272498

**Published:** 2014-01-29

**Authors:** Darren G. Craig, Patricia Lee, E. Anne Pryde, Ernest Hidalgo, Peter C. Hayes, Stephen J. Wigmore, Stuart J. Forbes, Kenneth J. Simpson

**Affiliations:** ^1^Gastroenterology Department, The James Cook University Hospital, Marton Road, Middlesbrough TS4 3BW, UK; ^2^Division of Clinical and Surgical Sciences, University of Edinburgh, Edinburgh EH16 4SB, UK; ^3^Adult and Paediatric Liver Services, St James's University Hospital, Leeds LS9 7TF, UK; ^4^MRC Centre for Inflammation Research, University of Edinburgh, Edinburgh EH16 4TJ, UK

## Abstract

*Background*. Small-for-size syndrome (SFSS) occurs in the presence of insufficient liver mass to maintain normal function after liver transplantation. Murine mortality following 85% hepatectomy can be reduced by the use of soluble receptor for advanced glycation end products (sRAGE) to scavenge damage-associated molecular patterns and prevent their engagement with membrane-bound RAGE. *Aims*. To explore serum levels of sRAGE, high-mobility group box-1 (HMGB1) protein, and other soluble inflammatory mediators in a fatal case of SFSS. *Methods*. Serum levels of HMGB1, sRAGE, IL-18, and other inflammatory mediators were measured by ELISA in a case of SFSS, and the results were compared with 8 patients with paracetamol-induced acute liver failure (ALF) and 6 healthy controls (HC). *Results*. HMGB1 levels were markedly higher in the SFSS patient (92.1 ng/mL) compared with the ALF patients (median (IQR) 11.4 (3.7–14.8) ng/mL) and HC (1.42 (1.38–1.56) ng/mL). In contrast, sRAGE levels were lower in the SFSS patient (1.88 ng/mL) compared with the ALF patients (3.53 (2.66–12.37) ng/mL) and were similar to HC levels (1.40 (1.23–1.89) ng/mL). *Conclusion*. These results suggest an imbalance between pro- and anti-inflammatory innate immune pathways in SFSS. Modulation of the HMGB1-RAGE axis may represent a future therapeutic avenue in this condition.

## 1. Introduction

The capacity for liver regeneration is finite, placing a restriction upon the minimum mass of liver tissue required to maintain hepatic function following split liver transplantation (LT) or liver resection. Small-for-size syndrome (SFSS) occurs in the presence of insufficient liver mass to maintain normal function and is characterised by severe graft dysfunction and increased ascites output [1]. The pathophysiology of SFSS is multifactorial, involving insufficient graft volume, poor graft quality, and excessive portal inflow [2]. Amplification of proinflammatory mediators in the remnant tissue is also recognised to play an important role in limiting liver regeneration [3]. Recent murine studies have suggested that a key pathway in this process involves the receptor for advanced glycation end products (RAGE), a cell-surface multiligand pattern recognition receptor linked with amplification of the innate inflammatory response to cell death. Engagement of membrane-bound RAGE with ligands such as high-mobility group box 1 (HMGB1) protein sustains inflammatory responses and promotes apoptosis in the hepatic remnant following massive hepatectomy [4]. Soluble RAGE (sRAGE), the truncated extracellular domain of RAGE, appears to act as a scavenger of RAGE ligands, thereby preventing membrane-bound RAGE activation. Infusion of sRAGE has been shown to significantly improve murine mortality following 85% hepatectomy [4], but, to date, HMGB1 and sRAGE expression have not been examined in human SFSS.

## 2. Case Description

A 65-year-old Caucasian female underwent cadaveric split LT for liver failure secondary to primary biliary cirrhosis (PBC). PBC was diagnosed 14 years earlier on the basis of a positive anti-mitochondrial antibody, cholestatic liver function tests, and a confirmatory liver biopsy. At the time of operation she weighed 47 kg with Child-Pugh and model for end stage liver disease scores of 8 and 16 points, respectively. She received a 500 g cadaveric left liver (segments I, II, III, and IV) graft, with a graft weight-to-recipient body weight ratio of 1.1%. The operation was uneventful, with a total blood volume loss of 680 mls. A routine postoperative ultrasound confirmed patent portal and hepatic artery inflow and patent hepatic venous outflow. Protocol immunosuppression consisted of tacrolimus, azathioprine, and prednisolone. By 7 days after LT, her clinical condition and transaminases had deteriorated markedly, with an alanine aminotransferase level rising from 69 IU/L preoperatively to 1035 IU/L. She underwent transjugular liver biopsy which demonstrated extensive haemorrhagic parenchymal, perivenular, and periportal necrosis consistent with portal hyperperfusion. Simultaneous hepatic venography demonstrated a gradient of 12 mmHg between the left hepatic vein and right atrium with no angiographically significant stenosis. Her clinical condition continued to deteriorate with rapid accumulation of ascites and she was reintubated and ventilated, and received significant inotropic and renal support. Repeat laparotomy demonstrated a congested liver with large volumes of intra-abdominal ascites. Due to continued clinical and biochemical deterioration she was listed for super urgent LT but died on the 11th day after LT before a second graft became available.

## 3. Methods

The study was prospectively approved by the Scotland “A” Research Ethics Committee. Informed consent or assent was obtained from all patients or the patient's nominated next of kin prior to study inclusion. Serum and plasma samples obtained from the peripheral circulation of the SFSS subject 10 days following LT were centrifuged at 1000 g for 15 minutes at 4°C within one hour following collection. Samples were immediately aliquoted and stored in polypropylene tubes at −80°C until analysis for soluble inflammatory mediators. As a pathological comparator group, samples obtained in an identical manner from 8 patients with fulminant acute liver failure (ALF) secondary to paracetamol (acetaminophen) poisoning were analysed. Four of these paracetamol overdose patients died whilst the other 4 patients underwent emergency LT. At the time of blood sampling, 7 of the 8 paracetamol patients were mechanically ventilated, and all 8 were receiving both inotropic support and continuous veno-veno haemofiltration. All 8 paracetamol patients were in grade 3-4 hepatic encephalopathy at the time of blood sampling.

### 3.1. HMGB1 Enzyme-Linked Immunosorbent Assay (ELISA)

Serum HMGB1 levels were measured using a commercial quantitative sandwich ELISA (Shino-Test Corporation, Japan) according to the manufacturer's instructions. Each sample was analysed in duplicate following appropriate dilutions. Results were determined from a standard curve prepared from 6 human HMGB1 standards ranging from 2.5 to 80 ng/mL. The coefficient of variation (CV) was 2.1%.

### 3.2. Soluble RAGE ELISA

Serum sRAGE levels were measured using a commercial quantitative sandwich ELISA (R&D Systems, Abingdon, UK). Each sample was analysed in duplicate following appropriate dilutions and results were obtained from a standard curve prepared from 7 human sRAGE standards ranging from 78 to 5000 pg/mL, with a CV of 1.4%.

### 3.3. IL-2 sR*α*, IL-18, and Neopterin ELISAs

Serum soluble IL-2 receptor alpha (IL-2 sR*α*), IL-18, and neopterin levels were measured using commercial quantitative sandwich ELISAs (R&D Systems Europe, Abingdon, UK; MBL International Corporation, Woburn, MA; and Demeditec Diagnostics, Kiel, Germany, resp.) according to the manufacturer's instructions. The CVs for these ELISAs were 1.0%, 2.7%, and 1.8%, respectively.

### 3.4. Serum IL-6 and IL-10

Measurements were performed using a cytometric bead array kit and software (BD Biosciences, San Jose, CA) according to the manufacturer's instructions, with cytokine analysis performed on a FACSArray flow cytometer (BD Biosciences, San Jose, CA).

## 4. Results

Levels of HMGB1 were markedly higher in the SFSS patient (92.1 ng/mL) compared with the paracetamol overdose patients (median (interquartile range) 11.4 (3.7–14.8) ng/mL, *n* = 8) and healthy controls (1.42 (1.38–1.56) ng/mL, *n* = 6). In contrast, sRAGE levels were lower in the SFSS patient (1.88 ng/mL) compared with the paracetamol overdose patients (3.53 (2.66–12.37) ng/mL, *n* = 8) and were similar to healthy controls (1.40 (1.23–1.89) ng/mL, *n* = 6). Further analysis of these data demonstrated considerably higher HMGB1: sRAGE levels in the SFSS patient (49.1) compared with the paracetamol overdose patients (1.7 (0.7–4.8), *n* = 8). The HMGB1 level remained at a similar level (82.7 ng/mL) in the SFSS patient on the 11th postoperative day.

### 4.1. IL-18 Levels

Following massive hepatic resection RAGE is upregulated on murine mononuclear phagocyte-derived dendritic cells (DCs) rather than hepatocytes [4]. DCs interact closely with natural killer cells to promote reciprocal maturation and activation, a process dependent upon secreted HMGB1, RAGE, and the cytokine interleukin (IL)-18 [5]. IL-18 levels in the SFSS patient were extremely elevated at 20880 pg/mL compared with both the paracetamol overdose cohort (527.7 (348.4–745.4) pg/mL, *n* = 8) and healthy controls (16.3 (10.5–90.9) pg/mL, *n* = 6).

### 4.2. Immune Activation

Overall activation of the lymphocyte and monocyte components of the immune response was assessed by measuring levels of soluble IL-2 sR*α*, a marker of T-cell activation, and neopterin, a marker of *γ*-interferon mediated macrophage activation. IL-2 R*α* levels were markedly increased in the SFSS patient (39.7 ng/mL) compared with the paracetamol overdose patients (4.4 (3.2–11.8) ng/mL, *n* = 8) and healthy controls (1.7 (1.3–1.8) ng/mL, *n* = 6). Likewise, neopterin levels were considerably higher in the SFSS patient (238.6 ng/mL) compared with the paracetamol-induced ALF patients (132.1 (83.9–161.7) ng/mL, *n* = 8) and healthy controls (11.4 (9.4–15.7) ng/mL, *n* = 6).

### 4.3. Regenerative and Anti-Inflammatory Cytokines

Massive hepatic resection may lead to impaired tissue regeneration and reduced anti-inflammatory responses to tissue injury. We measured levels of IL-6, an important regenerative cytokine normally upregulated following liver injury, and IL-10, a key anti-inflammatory cytokine, using a human inflammatory cytokine bead array. IL-6 levels were similar in both the SFSS patient (3518 pg/mL) and the paracetamol-induced ALF patients (4018 (1160–5000) pg/mL) whilst levels of IL-10 were only marginally raised in the SFSS patient (1350 pg/mL) compared with the paracetamol overdose cohort (556 (225–1969) pg/mL, *n* = 8).

## 5. Discussion

This study suggests that an imbalance in the HMGB1-RAGE axis may be involved in the pathogenesis of SFSS. Compared with a cohort of critically ill paracetamol overdose patients, all of whom died or required emergency LT, this SFSS patient exhibited eightfold higher HMGB1 levels, but lower levels of the HMGB1 scavenger sRAGE. Levels of IL-18, a key cytokine involved in DC and natural killer cell maturation, were extremely elevated in the SFSS patient compared with the paracetamol patients, as were IL-2 R*α* and neopterin, markers of T-cell and macrophage activation, respectively. However, levels of regenerative and anti-inflammatory cytokines were similar in both the SFSS and paracetamol patients. Importantly, both the SFSS and paracetamol patients had similar systemic organ failure assessment scores and organ support requirements, suggesting that the inflammatory dysregulation seen in the SFSS patient was not simply a consequence of greater multiorgan failure.

This case fulfils recognised definitions of SFSS given the onset of symptoms within a week of LT, the prolonged hyperbilirubinaemia, coagulopathy, encephalopathy, and absence of outflow obstruction [6]. However, we recognise that there are limitations to the conclusions that can be drawn from a single case, particularly since blood samples were only available from the 10th postoperative day. It is possible that the immune dysregulation seen in this case stems from cold or warm ischaemia at the time of LT. HMGB1 is rapidly released into the systemic circulation following liver reperfusion after LT, with HMGB1 levels correlating with the degree of graft steatosis and postoperative ALT levels [7]. However in the study by Ilmakunnas et al., HMGB1 levels fell rapidly within 1-2 hours of reperfusion after cold ischemia, suggesting that the high levels seen in our patient at 10 days postoperatively are unlikely to be directly related to preservation injury [7]. Interestingly, peripheral HMGB1 levels were considerably higher in our SFSS patient, using the same ELISA system, than those reported by Ilmakunnas et al. (range 2–40 ng/mL) following reperfusion in their post-LT cohort. The increased levels of HMGB1 may also reflect unrecognised systemic infection, particularly since there is impaired systemic immune function and decreased acute phase protein production following hepatectomy, and the ability of the host to fight systemic infection may also be impaired following a small-for-size graft [8, 9].

HMGB1, a regulatory nuclear protein involved in DNA transcription, is now recognized to have an important additional role as a damage-associated molecular pattern (DAMP) [10]. Following necrotic cell death hypoacetylated HMGB1 leaks from damaged cells where it can function as a danger signal to other cells and activate innate and adaptive immune responses [11]. Additionally, hyperacetylated HMGB1 is actively secreted by immune cells and can function as a cytokine [12, 13]. HMGB1 can activate a host of downstream effects including nuclear factor (NF)-*κ*B signalling, endothelial cell activation, and DC maturation [14]. Much attention has been focused upon HMGB1 as a late mediator of sepsis [10], but HMGB1 levels are also increased in a number of other acute and chronic inflammatory disorders [15, 16]. HMGB1 has been shown to play a role in experimental models of both paracetamol-induced hepatotoxicity [11, 17, 18] and ischaemia-reperfusion injury [19, 20]. The proinflammatory effects of HMGB1 may be enhanced by the formation of complexes with other inflammatory mediators such as IL-1*β*, nucleosomes, and lipopolysaccharide which then interact with a variety of receptors including RAGE [21]. Following HMGB1 stimulation, macrophages derived from RAGE knockout mice produce significantly lower amounts of proinflammatory and regenerative cytokines, but it is also important to note that HMGB1 can also act independently of RAGE via toll-like receptors −2, −4, and −9 and thus RAGE −/− mice are not completely protected from the effects of HMGB1 [22]. HMGB1 is also known to promote tissue regeneration and can modulate both endothelial and bone marrow stem cell function [16]. *In vivo*, HMGB1 can recruit mesoangioblasts to injured skeletal muscle and promote regeneration of skin wounds and cardiac muscle [23, 24]. It is noteworthy that the beneficial effects of HMGB1 upon stem cell migration and tissue repair are achieved at serum levels considerably lower than those seen in mice with septic shock, suggesting a possible threshold effect for HMGB1 beyond which tissue regeneration is overtaken by damage from infiltrating inflammatory cells.

Widespread application of cadaveric split LT or living donor LT could significantly improve the numbers of available organs for transplantation, but these options are currently limited by the need to provide sufficient functioning liver cell volume to the recipient. The use of a right hemi-hepatectomy from a liver donor places a healthy individual at significant operative risk, whilst a cadaveric graft rarely has sufficient liver volume to permit successful splitting to two adult recipients. Therefore, strategies to protect small grafts are urgently needed. Several animal studies have recognized enhanced innate immune response following small volume grafts [25–27], and recently membrane-bound RAGE was implicated in driving deleterious responses following massive murine liver resection [4]. RAGE can engage a variety of structurally diverse DAMPs, including HMGB1, released from dying cells. Engagement of membrane-bound RAGE with its various ligands sustains inflammatory responses in part through production of reactive oxygen intermediates and sustained activation of NF-*κ*B and mitogen-activated protein kinase pathways [28]. Modulation of HMGB1-RAGE binding could therefore influence cytokine production, cellular oxidant stress, and cell survival/proliferation. The potential beneficial effects of hepatic RAGE modulation have already been demonstrated experimentally in the context of paracetamol hepatotoxicity and ischaemia/reperfusion injury [29, 30], as well as following massive liver resection [4]. This suggests that RAGE may mediate a similar response to a number of different hepatic injuries and, as such, represents an attractive therapeutic target. However, exogenous sRAGE treatment produced only a modest benefit in a murine model of caecal ligation and puncture [31], and other caveats include a potential deleterious response when used during active bacterial infection [32].

The markedly increased levels of IL-18, neopterin, and IL-2 sR*α* in this case provide further evidence to support a role for macrophages and T cells in the pathogenesis of SFSS. A rat model utilizing small-for-size liver allografts demonstrated intense macrophage infiltration of the allograft by 72 hours, with increased IL-2 mRNA expression, suggesting that macrophages might accelerate cellular rejection in this syndrome in part through alloantigen presentation and adaptive immune activation [25]. Future animal studies should explore the temporal changes in HMGB1 levels following small-for-size allografting and determine the relationship between macrophage infiltration and HMGB1 expression in the liver remnant. In summary, SFSS remains a significant risk following split LT and major liver resection and this case sheds light upon the pathophysiology of this condition and, combined with the findings from previous animal studies in this area, suggests that modulation of the HMGBI-RAGE axis may represent a novel future therapeutic avenue.

## Abbreviations

LT: Liver transplantation
SFSS: Small-for-size syndrome
RAGE: Receptor for advanced glycation end products
HMGB1: High-mobility group box 1
sRAGE: Soluble RAGE
ALF: Acute liver failure
DC: Dendritic cell
IL: Interleukin
ELISA: Enzyme-linked immunosorbent assay
DAMP: Damage-associated molecular pattern
NF-*κ*B: Nuclear factor-*κ*B.
